# Integrative Analysis of Chromatin Accessibility and Transcriptional Landscape Identifies Key Genes During Muscle Development in Pigs

**DOI:** 10.3390/cells13242118

**Published:** 2024-12-20

**Authors:** Dongjie Zhang, Qian Zhang, Xiaoxu Wu, Liang Wang, Xiaohan Zhang, Di Liu, Xiuqin Yang

**Affiliations:** 1 Institute of Animal Husbandry, Heilongjiang Academy of Agricultural Sciences, Harbin 150086, China; djzhang8109@haas.cn (D.Z.); wlwl448@163.com (L.W.); 2 College of Animal Science and Technology, Northeast Agricultural University, Harbin 150030, China; 15169615521@163.com (Q.Z.); wxiaox2022@163.com (X.W.); zhangxiaohan21sdk@163.com (X.Z.)

**Keywords:** skeletal muscle, ATAC-seq, RNA-seq, transcription factor, dual-luciferase reporter analysis

## Abstract

Many efforts have been made to reveal the mechanisms underlying skeletal muscle development because of its importance in animals. However, knowledge on chromatin accessibility, a prerequisite for gene expression, remains limited. Here, dynamic changes in chromatin accessibility were analyzed in the skeletal muscles of Min pigs at the ages of 30, 90, and 210 d using an assay for transposase-accessible chromatin with high-throughput sequencing (ATAC-seq). A total of 16,301 differentially accessible regions (DARs) associated with 7455 genes were identified among three developmental stages. Seven out of eight DARs selected for a functional analysis were found to regulate reporter gene expression significantly (*p* < 0.05), indicating that DARs are active in gene expression. A total of 2219 differentially expressed genes (DEGs) were identified with RNA sequencing (RNA-seq). Through integrated analyses of ATAC-seq and RNA-seq data, 54 DEG_DAR_genes and 61 transcription factors (TFs) were characterized as critical for muscle development. Among them, Kruppel-like factor 5 (KLF5), targeted to 36 DEG_DAR_genes, was the most important TF. The effects of KLF5 on DEG_DAR_gene expression were then analyzed with molecular biology techniques. KLF5 was found to regulate *SLPI* (secretory leukocyte proteinase inhibitor) expression by directly binding to the promoter; KLF5 was also involved in *APOA1* (apolipoprotein A-I) expression through affecting the regulatory role of DAR located in the intron. These results indicate that the TFs identified were functional. Altogether, the chromatin accessibility region, TFs, and genes important for muscle development in Min pigs were identified. The results provide novel data for further revealing the mechanisms underlying the epigenetic regulation of muscle development.

## 1. Introduction

Skeletal muscle, comprising a mixture of stem cells, multinucleated myofibers, endothelial cells, adipocytes, neurocytes, etc., accounts for about 40% of animals’ body weight [1,2,3]. It determines not only the carcass meat yield but the meat quality. Therefore, skeletal muscle is directly related to the general economic benefits of swine production. It is of great importance for both breeders and producers to improve the growth and development of skeletal muscle. With the development of molecular biology and genetic engineering technology, the improvement of economic traits with molecular breeding methods, such as marker-assisted selection and genetically modified breeding, has become a research hotspot. In particular, for traits with low heritability such as muscle growth, molecular breeding has been demonstrated to be an effective tool.

The first step in molecular breeding is to reveal the mechanisms underlying trait formation. Many efforts have been made to clarify the genetic regulation of skeletal muscle formation in mammals. It has been demonstrated that the growth and development of skeletal muscle are precisely orchestrated by transcription factors (TFs), including paired box 3 (Pax3), Pax7, myogenic regulatory factors, and myocyte enhancer factor 2 [4,5,6]. At the same time, a large number of protein-coding genes, including myostatin, 5′-adenosine monophosphate-activated protein kinase, and fibroblast growth factor 19, have been identified as important regulators of muscle growth [7,8,9]. Additionally, noncoding RNAs (ncRNAs), including microRNAs, long ncRNAs, and circular RNAs, involved in the gene regulatory network, significantly contribute to the processes. Because of their intrinsic features in regulating gene expression, ncRNAs, especially miRNAs, have been found to be involved in almost every step of the biological process of muscle growth and development [10,11].

In recent years, increasing evidence has shown that epigenetic factors are important in the regulatory network of muscle growth. Chromatin accessibility, an important component of epigenomics, is directly associated with TF binding and thus controls the transcription of downstream genes, thereby playing crucial roles in various physiological and pathological processes. However, knowledge on chromatin accessibility during the postnatal growth and development of skeletal muscle in mammals is limited. An assay for transposase-accessible chromatin with high-throughput sequencing (ATAC-seq) is a useful tool for analyzing chromatin accessibility [12,13,14]. Through an integrated analysis of ATAC-seq and RNA-seq data, researchers have revealed several core genes and pathways controlling muscle development in Landrace pigs [15]. However, as an epigenetic phenomenon, chromatin accessibility is affected by multiple factors, including pig breed and environment. Studies have shown that there are many differences in chromatin accessibility between Luchuan and Duroc pigs [16]. Min pig is a local pig breed unique to the cold northeast of China. Compared with commercial pig breeds such as Landrace and Duroc, Min pig has delicious meat but slow growth [17,18]. We hypothesized that Min pig had a specific chromatin accessibility profile during muscle development. To characterize the changes in chromatin accessibility and TFs important for muscle development in Min pigs, ATAC-seq and RNA sequencing (RNA-seq) were performed on the muscles of the pigs at three representative time points of the nursery and growth/finish phases. The results will contribute to further revealing the molecular mechanism underlying muscle growth and development and to broadening our knowledge on epigenetic regulation during muscle formation.

## 2. Materials and Methods

### 2.1. Animals and Nucleic Acids

Min pigs used were male and provided by the Institute of Animal Husbandry, Heilongjiang Academy of Agricultural Sciences, Harbin, China. The longissimus dorsi muscles were collected from the pigs at 30, 90, and 210 d old, each with three individuals (n = 9). At each developmental stage, three full-sib barrows, reared under the same conditions and allowed to feed ad libitum, were slaughtered, and the muscles were collected immediately. The samples were snap-frozen in liquid nitrogen and then stored at −80 °C until use. Total RNA was extracted with TRIzol reagent (Invitrogen, Carlsbad, CA, USA) based on the manufacturer’s instructions. DNA was isolated with DNAiso Reagent (TaKaRa, Dalian, China) according to the manufacturer’s instructions. The quality and concentration of nucleic acids were measured with a NanoDrop One (Thermo Scientific, Wilmington, DE, USA). All animal treatments were approved by the Laboratory Animal Welfare and Ethics Committee of Northeast Agricultural University.

### 2.2. ATAC-Seq Library Construction and Sequencing

ATAC-seq was performed on the porcine muscles at three developmental stages, each in triplicate. A library was constructed using a TruePrep DNA Library Prep Kit V2 for Illumina^®^ (Vazyme, Nanjing, China). Briefly, the nuclei were isolated via density gradient centrifugation and purified with a MinElute PCR Purification Kit (Qiagen, Hilden, Germany). Transposition was performed at 37 °C for 30 min. After purification, the DNA was PCR-amplified with a TruePrep^TM^ Index Kit V2 for Illumina^®^ (Vazyme) using 10 cycles. The products were purified with magnetic beads. Sequencing was performed using the Illumina NovaSeq 6000 platform by Biomarker Technologies (Wuhan, China).

### 2.3. ATAC-Seq Data Analysis

Raw data were filtered with cutadapt (v1.8.3) to obtain high-quality clean reads [19]. The clean reads were mapped to a reference genome (*S. scrofa* 11.1_release109) using Bowtie 2 (v2.2.4) [20]. The density distribution was mapped within 3 kb upstream and downstream of the transcription start sites (TSSs) of the gene using DeepTools (v3.2.0) [21]. Peak calling was performed with MACS2 (v2.2.7.1) software, and a false detection rate (FDR) < 0.05 was set as the threshold [22]. Peaks were annotated to the genomic regions and assigned to the nearest gene with the ChIPseeker R package (v1.2.6) [23]. The motif was identified with MEME-ChIP (v4.11.2) and aligned against a known motif database with the Tomtom motif database-scanning algorithm to predict TFs [24]. Differentially accessible chromatin regions (DARs) were identified with DEseq2 (v1.30.1) using a threshold of absolute log2 fold-change (FC) ≥ 1 and FDR < 0.05 [25]. The functional enrichment of genes was performed using the Gene Ontology (GO) and Kyoto Encyclopedia of Genes and Genomes (KEGG) databases with the R package clusterProfiler (v4.03) [26,27].

### 2.4. RNA-Seq Data Analysis

We obtained RNA-seq data on the porcine muscles at five developmental stages, including on the same nine samples used for ATAC-seq. The data have been submitted to Genome Sequence Archive (GSA; https://ngdc.cncb.ac.cn/gsa/s/a6TPc6SR; accession number: CRA013591, submitted on 24 October 2024) Here, the data on the nine samples were extracted and analyzed to determine the expression changes in genes during the different stages. We first identified differentially expressed genes (DEGs) using DESeq2 (v1.30.1), with a criterion of absolute log2 (FC) ≥ 1 and FDR < 0.05, in the pig groups of 30_vs_90, 30_vs_210, and 90_vs_210. A multivolcano plot was constructed with OmicShare tools (https://www.omicshare.com/tools (accessed on 1 September 2024). Venn and heatmap diagrams were plotted using online tools (http://www.bioinformatics.com.cn/ (accessed on 1 September 2024). Mfuzz (http://mfuzz.sysbiolab.eu (accessed on 5 September 2024) was used to perform gene clustering based on the DEG expression levels. The GO and KEGG enrichments were analyzed as described above.

### 2.5. Cell Culture and Transfection

Porcine skeletal muscle satellite cells (PMSCs), provided by MINGZHOUBIO (Ningbo, China), were cultured in Dulbecco’s Modified Eagle’s Medium (DMEM)/Nutrient Mixture F-12 (F12) containing 10% fetal bovine serum (FBS; MINGZHOUBIO), 1% penicillin–streptomycin (Invitrogen), and 1% growth factor (MINGZHOUBIO). PK-15 and C2C12 cells were cultured in DMEM/F12. HEK-293T cells were cultured with high-glucose DMEM. The media for PK-15, C2C12, and HEK-293T were supplemented with 10% FBS (OPCEL, Huhehaote, China) and 1% penicillin–streptomycin (Invitrogen). The cells were cultured at 37 °C with 5% CO_2_, and the medium was changed every 48 h. Transfection was performed with Lipofectamine 2000 (Invitrogen), according to the manufacturer’s instructions.

### 2.6. Dual-Luciferase Reporter Analysis

A pGL3 basic or promoter vector (Promega, Fitchburg, WI, USA) was selected to construct luciferase reporter genes according to the localization of the inserted fragments in the corresponding genes; that is, the fragments located on the promoter were inserted into the pGL3 basic vector at the *Hin*dIII site, while those located downstream of the gene were inserted into the pGL3 promoter vector at the X*ba*I site. Fragments were amplified with genomic DNA template using *Premix* Taq™ (TaKaRa) and then ligated to the vector using a ClonExpress II One Step Cloning Kit (Vazyme). Deleting of putative motif for Kruppel-like factor 5 (KLF5) was conducted with overlapping extension PCR as described previously [28]. Each reporter gene was cotransfected into PK-15 cells with pRL-TK, a *Renilla* luciferase reporter. At 48 h post-transfection, luciferase activities were measured with a dual-luciferase reporter gene assay kit (Beyotime, Shanghai, China). The relative luciferase activities of firefly to the *Renilla* luciferase value were calculated. The primers used for reporter construction are shown in Table S1.

### 2.7. Western Blotting

Plasmids overexpressing *KLF5* were constructed with an improved pCAGGS plasmid provided by Dr. Honglin Jia. The coding sequence (CDS) of *KLF5* was first amplified with a cDNA template obtained from the muscles of Min pigs and PrimeSTAR HS DNA polymerase (TaKaRa), and then sequences encoding three HA peptides were added to the 5′ end of the CDS using overlapping-extension PCR. The resulting products were inserted into the improved pCAGGS at the *Sam*I site using a ClonExpress II One Step Cloning Kit (Vazyme). Western Blotting was carried out as described previously [28]. Briefly, the recombinant plasmids and empty vector, used as a control, were transiently transfected into PK-15 cells. After culturing for 48 h, the cells were collected to isolate proteins with a RIPA buffer (Beyotime) containing a protease inhibitor (Invitrogen). A BCA protein assay kit (Beyotime) was used for protein quantification. Furthermore, 20~25 µg total protein was used for incubation with anti-HA tag (1:5000 dilution; Abmart, Shanghai, China) and anti-β-tubulin (1:1000 dilution; Abmart) primary antibodies. Goat antimouse IgG (1:20,000 dilution; LI-COR, Lincoln, NE, USA) was used as a secondary antibody. The results were visualized with UVP ChemStudioTM PLUS touch (Analytik Jena, Upland, CA, USA).

### 2.8. Real-Time Quantitative PCR

Reverse transcription (RT) was carried out using a PrimeScript^TM^ RT Reagent Kit (TaKaRa) based on the manufacturer’s instructions. Real-time quantitative PCR (qPCR) was performed using ChamQ Universal SYBR qPCR Master Mix (Vazyme) with β-actin as a reference, and the data were measured using the 2^−ΔΔCt^ method [29]. To measure the effects of KLF5 on secretory leukocyte proteinase inhibitor (SLPI) expression, plasmids overexpressing *KLF5* were transfected into PK-15 and PMSC cells. At 24 h post-transfection, the cells were collected for mRNA isolation, and then cDNA was obtained as described above. Primer information is shown in Table S1.

### 2.9. Electrophoretic Mobility Shift Assay

An electrophoretic mobility shift assay (EMSA) was performed using a chemiluminescence kit (Beyotime) as previously described [28]. Briefly, nuclear extracts were obtained from HEK-293T cells using the kit (Solarbio, Beijing, China). Biotin-labeled and unlabeled probes, used for specific competitors for KLF5-specific binding, and mutant competitors were provided by General Biol (Hefei, China). The labeled probes were incubated with nuclear extracts alone or together with the mutant competitor for 20 min. As for coincubating the labeled probes with the specific competitor, the specific competitor was incubated with nuclear extracts for 10 min before the labeled probes were added. The mixture was electrophoresed on 6.5% polyacrylamide gel, transferred to a nylon membrane (Beyotime), and visualized on an Azure c300 Gel Imaging System (Zzure Biosystems, Dublin, CA, USA). The probe sequences are given in Table S1.

### 2.10. Chromatin Immunoprecipitation–Real-Time Quantitative PCR

Chromatin immunoprecipitation–real-time quantitative PCR (ChIP-qPCR) was carried out with a SimpleChIP^®^ Enzymatic Chromatin IP Kit (Cell Signaling Technology, Danvers, MA, USA) according to the manufacturer’s instructions. Briefly, plasmids overexpressing *KLF5* were transfected into PK-15 cells for 48 h. A total of 2 × 10^7^ cells were crosslinked in 1% formaldehyde for 10 min at room temperature, and 10 × glycine solution was used to terminate the crosslinking reaction. After washing with ice-cold phosphate-buffered saline (PBS), the cells were collected with 2 mL PBS + PIC (protease inhibitor cocktail) and centrifuged at 4 °C, 2000× *g* for 5 min. The nuclear pellet was shivered to ~150–900 bp in length with an ultrasonic homogenizer. The DNA was then incubated with anti-HA tag (Proteintech, Wuhan, China). Finally, qPCR was performed to quantify the immunoprecipitated DNA. The primers used for ChIP-qPCR are listed in Table S1.

### 2.11. Statistical Analysis

All the experiments were independently performed in triplicate. An unpaired *t*-test was used to assess the statistical significance of the differences between groups, while a one-way ANOVA was used to assess that among multiple groups. Data are shown as means ± standard deviations. GraphPad Prism 9.0 was used to analyze the data, with default parameters.

## 3. Results

### 3.1. Overview of Skeletal Muscle ATAC-Seq Data

**Figure 1:**
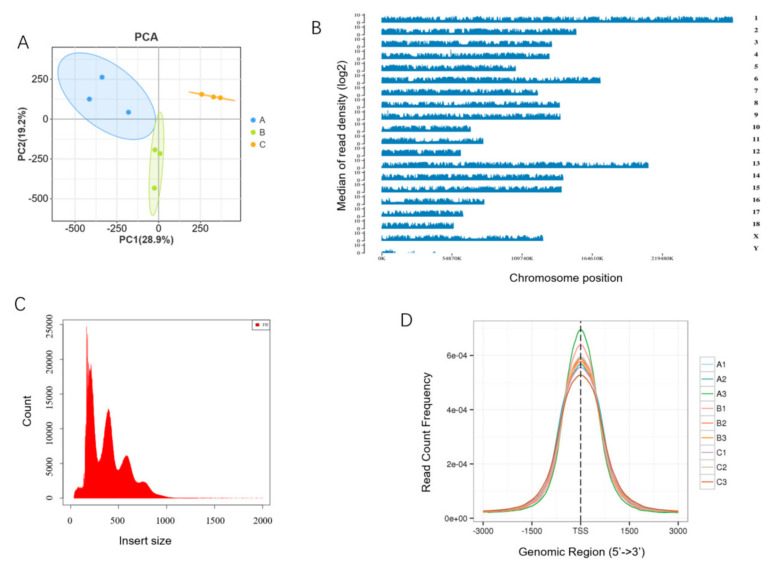
Quality control of ATAC-seq. (**A**) Plot of principle component analysis; (**B**) genome-wide distribution of read coverage, exemplified with A1; (**C**) insert size histogram for all reads, exemplified with A1; (**D**) distribution of the number of peaks in the TSS region. A, B, and C in the legend indicate samples from 30-, 90-, and 210-day-old pigs, respectively, the same as below.

To reveal the epigenetic regulatory mechanisms underlying the growth and development of skeletal muscle in Min pigs, ATAC-seq was performed on longissimus dorsi muscles obtained from 30-, 90-, and 210-day-old pigs, each in triplicate. A total of 1,087,247,155 clean reads, with an average of 120,805,239.4 per sample, were obtained from nine samples, comprising at least 94.41% Quality 20 (Q20) reads and 86.46% Q30 reads. In each sample, > 95.2% clean reads were mapped to a reference genome (*S. scrofa* 11.1_release109), and > 79.56% clean reads were uniquely mapped (Table S2). A principal component analysis (PCA) showed good repeatability of the triplicates in the same stage and separation between the three stages (Figure 1A). The uniquely mapped reads were ubiquitously distributed in all chromosomes (Figure 1B). The length of the inserted fragments was mainly about 100–200 bp in each library, indicating that nucleosome-free and single-nucleosome fragments were dominant (Figure 1C). The density distribution mapping of the reads showed a significant enrichment of chromatin accessible regions within a 3 kb area of the TSSs (Figure 1D). These results indicate that the ATAC-seq data obtained were of high quality and could be used for further analyses.

### 3.2. Chromatin Accessibility in Skeletal Muscles

**Figure 2:**
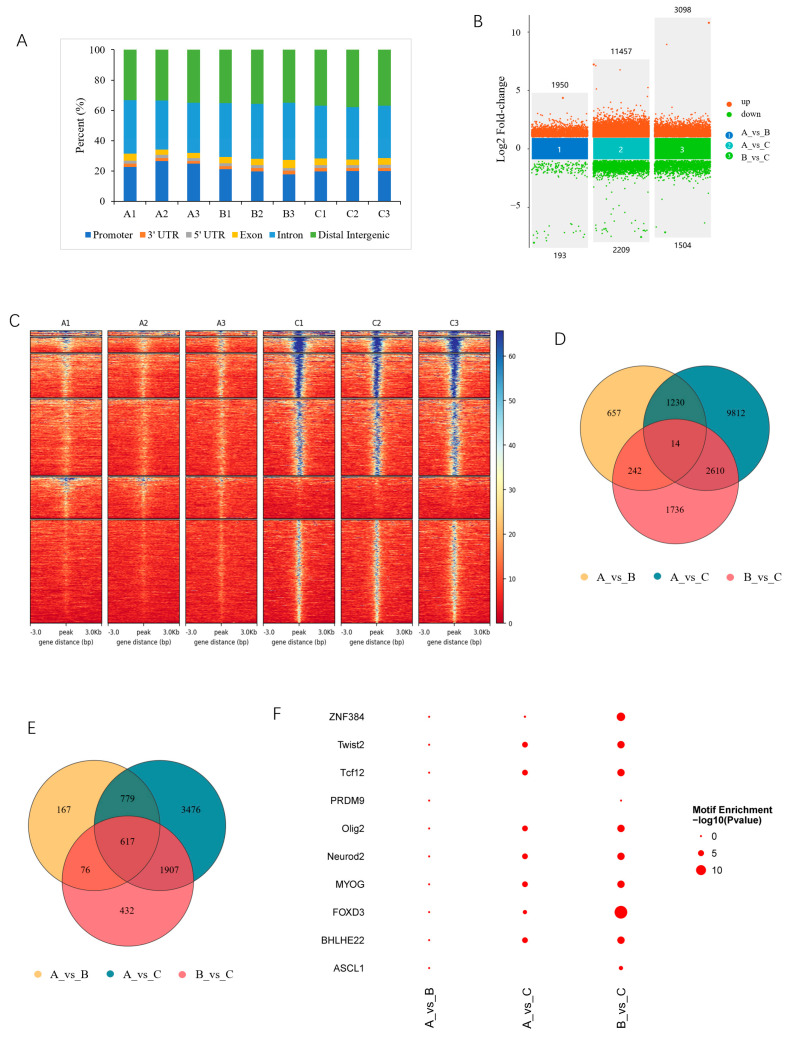
Characterization of differentially accessible regions (DARs). (**A**) Distribution of all peaks in the functional region of genes; (**B**) multivolcano plot of DARs; (**C**) clustering heatmaps of DARs, exemplified with group 30-vs-210; (**D**) Venn diagram of DARs; (**E**) Venn diagram of DAR-associated genes (DAR_genes); (**F**) top 10 transcription factors enriched in DARs.

A total of 2,233,422 peaks were obtained, with an average of 248,158 in each library (Table S2). A genome-wide distribution analysis showed that a considerable number of peaks were located in promoter regions (Figure 2A). Based on the thresholds of absolute log2(FC) ≥ 1 and FDR < 0.05, a total of 16,301 DARs were identified among the three developmental stages. Among them, there were 2143 DARs, comprising 1950 increased and 193 decreased in 90- compared with 30-day-old pigs (group 30_vs_90-d); 13,666 DARs, comprising 11,457 increased and 2209 decreased in 210- compared with 30-day-old pigs (group 30_vs_210-d); and 4602 DARs, comprising 3098 increased and 1504 decreased in 210- compared with 90-day-old pigs (group 90_vs_210-d) (Figure 2B and Table S3). A heatmap analysis revealed a consistent expression of DARs within the same groups (Figure 2C), similarly to the PCA results (Figure 1A), further confirming the reliability of ATAC-seq. Furthermore, 14 DARs were shared between all comparison groups (Figure 2D and Table S3). DARs were associated with 7455 genes, and among them, 1640, 6780, and 3033 genes were found in the 30_vs_90-d, 30_vs_210-d, and 90_vs_210-d groups, respectively. Additionally, 617 genes were shared among all three comparison groups (Figure 2E and Table S3). The TFs enriched in DARs are involved in various physiological processes, such as myogenesis and neurogenesis (Figure 2F).

**Figure 3:**
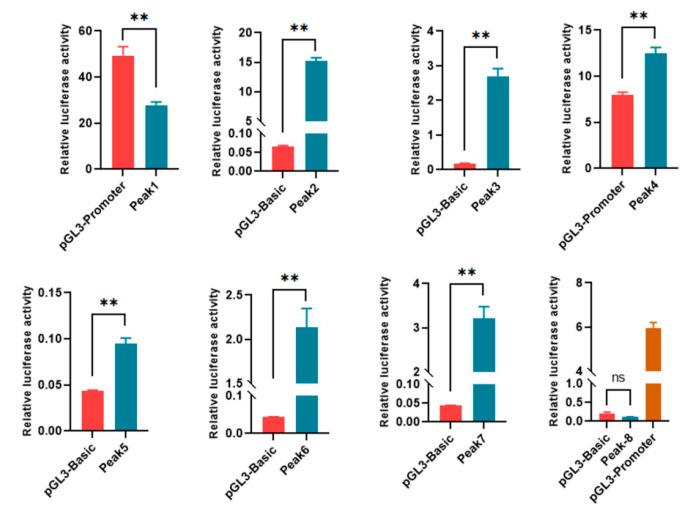
Functional identification of DARs with dual-luciferase reporter analysis. Peak 8 was inserted into pGL3 basic, and pGL3 promoter was used as a positive control. ns, *p* > 0.05; **, *p* < 0.01.

To analyze the effects of DARs on gene expression, eight of the DARs mapped 3 kb upstream and downstream of the TSSs were selected for dual-luciferase reporter analyses (Table S4). Among them, Peaks 1 and 4 were located on the 3′ end and intron of the associated genes, respectively; thus, they were inserted into pGL3 promoter vectors. The remaining peaks were in the promoter regions of the genes, and thus, reporter genes were constructed with a pGL3 basic backbone. Seven out of the eight DARs were demonstrated to regulate the expression of the luciferase gene, indicating the involvement of DARs in regulating the expression of associated genes (Figure 3).

### 3.3. Differentially Expressed Genes Among Skeletal Muscles

**Figure 4:**
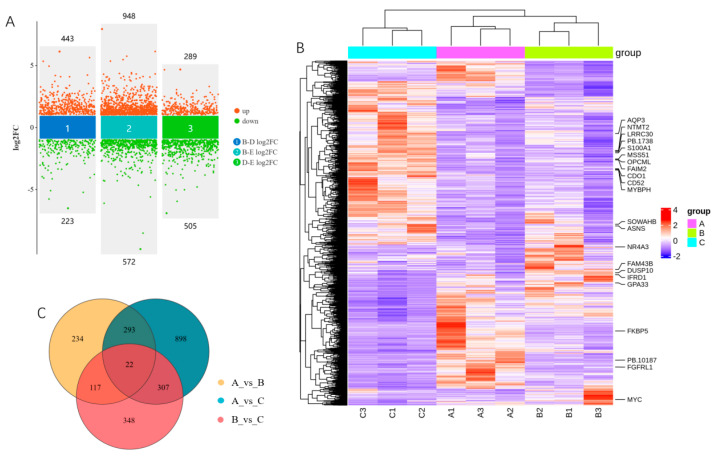

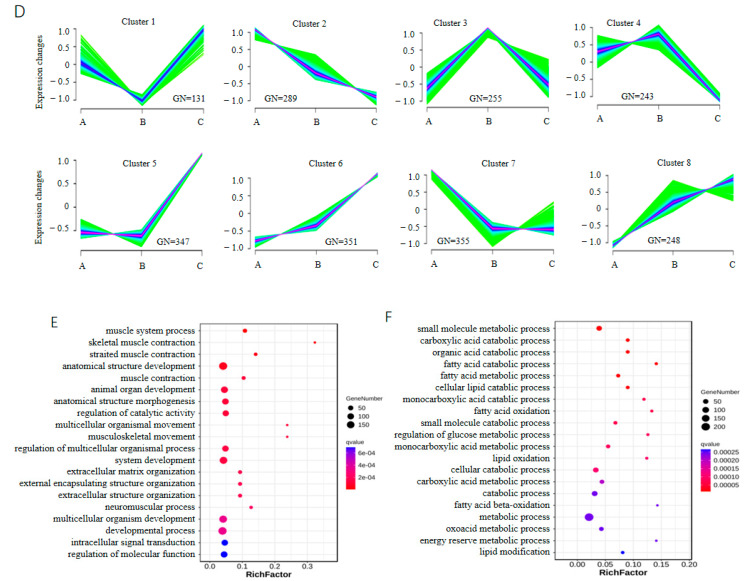
Characterization of differentially expressed genes (DEGs) with RNA-seq. (**A**) Multivolcano plot of DEGs; (**B**) heatmap of DEGs. Genes shared by all three comparison groups are shown on the right. (**C**) Venn diagram of DEGs; (**D**) clustering of DEGs, where GN indicates gene number; (**E**) top 20 biological process terms enriched by DEGs in clusters 6 and 8; (**F**) top 20 biological process terms enriched by DEGs in cluster 7.

According to the threshold set, there were 666 DEGs, comprising 443 upregulated and 223 downregulated, in group 30_vs_90-d; 1520 DEGs, comprising 947 upregulated and 572 downregulated, in group 30_vs_210-d; and 794 DEGs, comprising 289 upregulated and 505 downregulated, in group 90_vs_210-d (Figure 4A,B; Table S5). A total of 2219 unique DEGs were identified in the three comparison groups, and 22 DEGs were shared between them, including myosin binding protein H; MYC; Fas apoptotic inhibitory molecule 2; mitochondrial translational activator; and nuclear receptor subfamily 4, group A, member 3, etc. (Figure 4B,C; Table S5).

**Figure 5:**
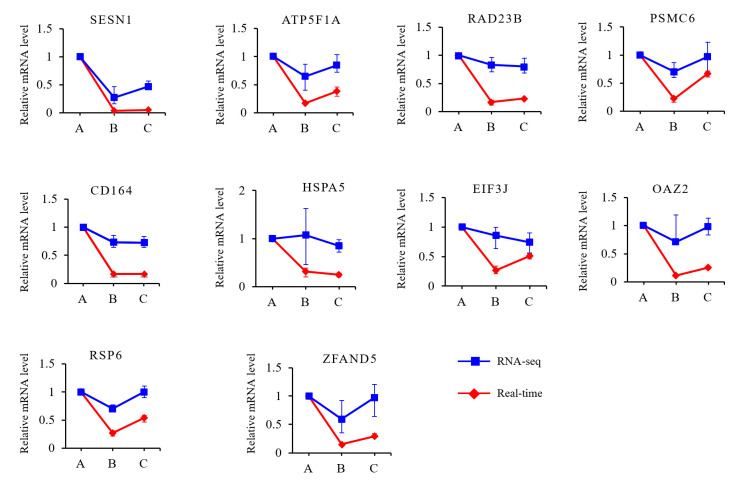
Validation of RNA-seq data with real-time quantitative PCR.

Sequential analyses were performed on the 2219 DEGs to reveal the expression changes during the growth and development of the muscles. The DEGs were grouped into eight clusters (Figure 4D and Table S5). The expression of the genes in clusters 6 and 8 continuously increased as development progressed, while that in cluster 2 decreased. GO enrichment showed that the genes in clusters 6 and 8 were mainly enriched in biological process (BP) terms associated with muscle development, including muscle system process, skeletal muscle contraction, striated muscle contraction, and musculoskeletal movement (Figure 4E). The genes in cluster 2 were mainly enriched in BP terms associated with metabolic processes, such as organic substance metabolic process, primary metabolic process, and cellular metabolic process (Supplementary Figure S1). In cluster 7, the expression of genes strongly decreased from 30 to 90 d and then remained steady from 90 to 210 d. The genes in cluster 7 were mainly enriched in BP terms associated with fat catabolism, such as fatty acid catabolic process, fatty acid oxidation, and carboxylic acid catabolic process (Figure 4F), which was consistent with the pattern of adipose development processes in Min pigs. Among the genes expressed in all three developmental stages, ten were selected for validation with qPCR. Similar trends in expression changes among the three stages were observed (Figure 5).

### 3.4. Integrated Analysis of Chromatin Accessibility and Gene Expression

**Figure 6:**
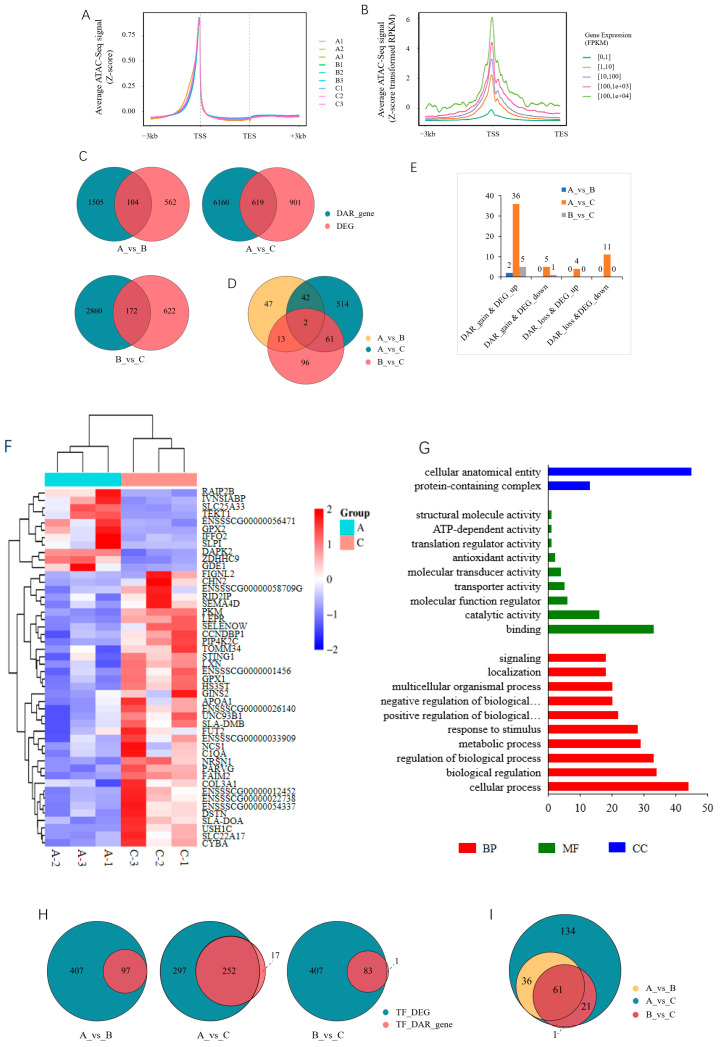
Integrated analysis of ATAC-seq and RNA-seq. (**A**) Distribution of ATAC-seq signal along gene body; (**B**) relationship between ATAC-seq signal and gene expression level, with a representative sample (A1@ATAC-seq_vs_A1@RNA-seq) given; (**C**) Venn diagram of DEGs and DAR_genes in each comparison group; (**D**) Venn diagram of interaction results of DEGs and DAR_genes among the three comparison groups; (**E**) identification of DEGs containing DARs in the promoter (DEG_DAR_genes); (**F**) heatmap of DEG_DAR_genes in 30_vs_210-d group; (**G**) top 10 GO terms enriched by DEG_DAR_genes in the three comparison groups. BP, biological process. MF, molecular function. CC, cellular component. “…” indicated pathway. (**H**) Venn diagram of TFs predicted in the promoter of DEG_DAR_genes; (**I**) Venn diagram of interaction results of TFs among the three comparison groups.

An integrated analysis was performed on the ATAC-seq and RNA-seq data to explore the relationship between chromatin accessibility and gene expression. The ATAC-seq signal was concentrated in the gene body, and its distribution was positively correlated with the gene expression level (Figure 6A,B). DAR-associated genes first intersected with DEGs, and 104, 619, and 172 genes were common in the 30_vs_90-d, 30_vs_210-d, and 90_vs_210-d comparison groups, respectively; among them, 2 were shared by all three comparison groups (Figure 6C,D; Table S6). Then, DEGs containing DARs in the promoter (DEG_DAR_gene) were screened out, and a total of 54 unique genes were identified among the three comparison groups, with the most, 48, being in the 30_vs_210-d group (Figure 6E,F; Table S7). The GO enrichment analyses showed that the 54 genes were involved in all three categories, and cellular process, biological regulation, metabolic process, and developmental process were among the top 10 terms in the biological process category (Figure 6G). The TFs were then predicted in the promoter regions of these DEG_DAR_genes, and 97, 252, and 83 TFs were identified in the 30_vs_90-d, 30_vs_210-d, and 90_vs_210-d groups, respectively. A total of 61 TFs were shared by all three comparison groups (Figure 6H and Supplementary Table S8).

### 3.5. TF Validation

**Figure 7:**
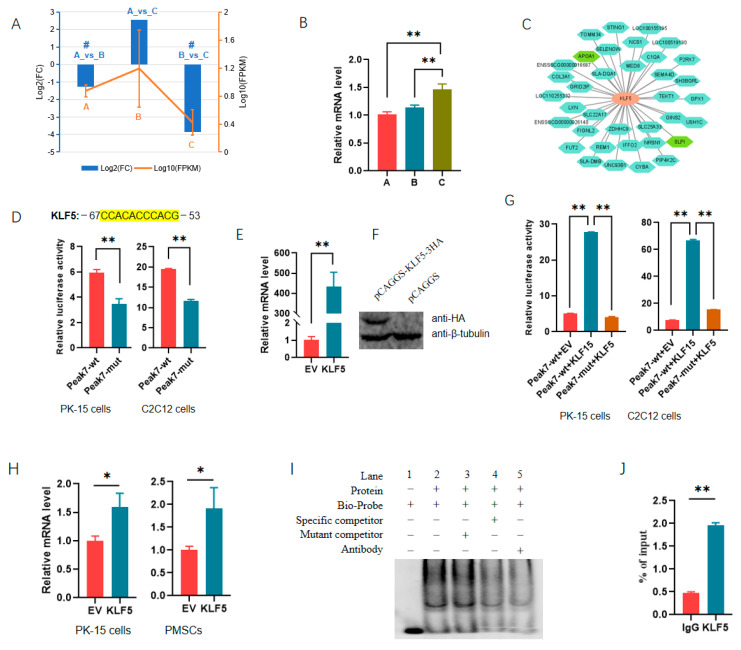
Transcription factor KLF5 regulates the expression of *SLPI* by directly binding to the promoter. (**A**) Expression profile of *KLF5* during three developmental stages, as revealed by RNA-seq. # indicates that *KLF5* was differentially expressed in the comparison group. A, B, and C indicate 30-, 60-, and 90-day-old pigs. (**B**) Validation of *KLF5* with qPCR in muscles at different stages. The level in 30 d old pigs was set to 1. (**C**) DEG_DAR_genes targeted by KLF5; (**D**) effect of the putative binding site for KLF5 on the expression of reporter gene containing Peak 7. The putative site for KLF5 is shown above. wt, wild-type reporter gene. mut, mutant-type reporter gene. In mut-type reporter gene, the binding sites were all deleted. (**E**,**F**) Efficiencies of plasmids overexpressing *KLF5* at mRNA (**E**) and protein levels (**F**); (**G**) effect of ectopic *KLF5* on the activities of reporter genes containing Peak 7; (**H**) ectopic *KLF5* on endogenous expression of *SLPI* gene. PMSC indicates porcine skeletal muscle satellite cells. (**I**) EMSA revealed KLF5 bound to the promoter of *SLPI* through the putative site; (**J**) ChIP-qPCR assay validated the putative binding site for KLF5 in the promoter of *SLPI*. *, *p* < 0.05; **, *p* < 0.01.

Among the 61 TFs, KLF5 and ZNF148 had the most target genes, with each of them targeted to the promoter of 36 DEG_DAR_genes (Supplementary Table S8). KLF5 was identified as a DEG in both the 30_vs_210 and 90_vs_210 groups, and its expression changed dramatically among the three stages (Figure 7A,B), suggesting that it is important for muscle development in Min pigs. Peak 7, having been shown to regulate the expression of reporter genes (Figure 3), was found to be a promoter of secretory leukocyte peptidase inhibitor (SLPI). *SLPI* was among the 36 DEG_DAR_genes targeted by KLF5 (Figure 7C). The regulatory role of KLF5 in *SLPI* expression was analyzed using molecular biology techniques.

We first deleted the putative binding motif of KLF5 in the reporter gene containing Peak 7, and we found that this resulted in a significant decrease in luciferase activity in both PK-15 and C2C12 cells (Figure 7D). Plasmids overexpressing *KLF5* were constructed successfully (Figure 7E,F). The overexpression of *KLF5* significantly increased luciferase activity, while the deletion of the motif abolished the promoting effects of ectopic KLF5 on the expression of the reporter gene (Figure 7G). Additionally, ectopic KLF5 significantly increased the mRNA level of the *SLPI* gene in both PK-15 and PMSCs (Figure 7H). An EMSA showed that the biotin-labeled probe could generate DNA–protein complexes with nuclear extracts, and the mutant competitor had no effect on the complex. At the same time, the complexes were significantly weakened by the specific competitor. Furthermore, the addition of an antibody reduced the complexes robustly (Figure 7I). ChIP-qPCR also indicated that KLF5 had significant enrichments in the Peak 7 region (Figure 7J). These results confirm that KLF5 regulated the transcription of *SLPI* by directly binding to the promoter, indicating the reliability of the TFs identified here.

**Figure 8:**
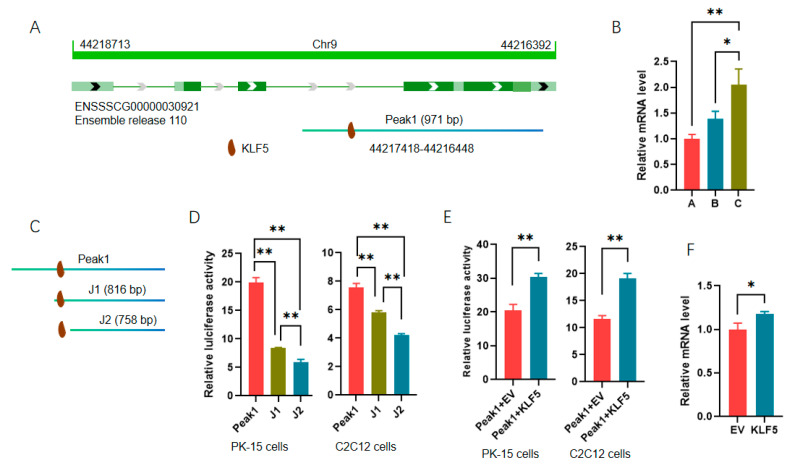
Transcription factor KLF5 is involved in the regulation of *APOA1* expression. (**A**) Schematic diagram of *APOA1* gene, associated DAR, and putative TFs; (**B**) validation of *APOA1* with qPCR in muscles at different stages. The level in 30 d old pigs was set to 1. A, B, and C indicate 30-, 60-, and 90-day-old pigs. (**C**) Schematic diagram of truncated reporter genes; (**D**) relative luciferase activities of truncated reporter genes; (**E**) effects of ectopic KLF5 on the activities of reporter genes containing Peak 1; (**F**) effects of ectopic KLF5 on the mRNA level of *APOA1* gene in PMSCs. *, *p* < 0.05; **, *p* < 0.01.

Additionally, Peak 1 was identified as a DAR of the apolipoprotein A-I (APOA1) gene, also a DEG_DAR_gene targeted by KLF5 (Figure 7C). Sequence analyses showed that *APOA1* was located on chromosome 9 and encoded by the antisense strand, and Peak 1 covered the middle part of intron 3 to the 3′ end of the gene. It is interesting that there is a putative binding site for KLF5 in the intron region of *APOA1* (Figure 8A). To analyze the regulatory role of KLF5 in *APOA1* expression, we first validated the RNA-seq data with qPCR, and consistent results were obtained (Figure 8B). Additionally, two 5′ truncated fragments of Peak 1 were amplified according to the motif prediction results, and the reporter genes were constructed (Figure 8C). Each of the truncated fragments was found to significantly decrease the luciferase activity in both PK-15 and C2C12 cells (Figure 8D). Compared with the truncated fragment J1, only the putative site of KLF5 was absent from J2, indicating that the site is functional. Plasmids overexpressing *KLF5* were then cotransfected into cells with Peak 1 to characterize the role of KLF5 in *APOA1* expression, and the promoting effects of KLF5 were confirmed in both PK-15 and C2C12 cells (Figure 8E). Furthermore, ectopic *KLF5* significantly increased the expression of *APOA1* in PMSCs at mRNA level (Figure 8F). The results show that KLF5 could regulate the expression of *APOA1*, but the underlying mechanisms need to be further analyzed.

## 4. Discussion

Chromatin accessibility, an important epigenetic regulator, directly determines the recognition and binding of TFs, and, thus, it is crucial for gene expression. However, knowledge on the dynamic changes in chromatin accessibility in skeletal muscle development in mammals remains limited. Here, we generated profiles of chromatin accessibility, as well as gene expression, in the skeletal muscles of Min pigs at three developmental stages using ATAC-seq and RNA-seq. Through integrated analyses of chromatin accessibility and gene expression, key genes in the growth and development of skeletal muscle in Min pigs were identified. Importantly, the TFs of the genes were characterized concurrently. The results will contribute to fully revealing the mechanisms underlying skeletal muscle growth and development.

Skeletal muscle development, directly associated with meat production and quality in agricultural animals, is a complicated process and precisely orchestrated by a strictly regulated gene network. Both genetic and epigenetic factors are important in ensuring the exact expression of genes. In the past few decades, efforts have mainly focused on genetic factors to reveal the mechanisms underlying the growth and development of skeletal muscle [30,31,32]. Research on epigenetic factors is much less, especially on chromatin accessibility; to date, only a few studies have been conducted on chromatin accessibility [15,33,34]. In this study, the peaks obtained were distributed universally in the genome, indicating a good coverage of ATAC-seq. A great number of DARs were identified in three comparison groups: 30_vs_90-d, 30_vs_210-d, and 90_vs_210-d. However, only 14 DARs were shared by the three groups, and a large number of DARs were specific to one comparison group, consistently with the distribution of DEGs among the comparison groups.

Through a motif analysis, it was found that various TFs were enriched in the DARs. Among the top 10 enriched TFs were myogenic lineage and neurogenic lineage TFs, MyoG, and NeuroD2. As one of the four myogenic regulatory factors, MyoG controls the terminal differentiation of myogenic cells, and its absence results in poor skeletal muscle development and animal death at birth [35,36,37]. NeuroD2, a master regulator of neurogenesis, has been demonstrated to be a lineage determining TFs, and it can transdifferentiate cells to neurons [38,39,40]. Among the remaining top 10 TFs, some have been determined to be involved in muscle development, such as FOXD3 and Twist [41,42,43], while some have not yet been mentioned in the process, such as Tcf12 and PRDM9. Additionally, analyses of the dual-luciferase reporter gene confirmed that DARs may regulate gene expression, indicating from one perspective the potential role of the TFs identified. The diversity of the TFs obtained here further indicates the complexity of skeletal muscle growth and development. Feng et al. [15] analyzed the chromatin accessibility profile during the muscle growth and development of postnatal Landrace pigs. There are many differences between their results and ours in terms of the peak distribution and TF enrichment. The Min pig is a local Chinese pig breed different from Landrace pigs in terms of meat quality and muscle development characteristics [17,18]. Additionally, the time points examined slightly differ between the two studies.

Using RNA-seq, the gene expression profiles of the muscles in the three developmental stages were identified, and DEGs were obtained. As observed in the DARs, the number of DEGs in the 30_vs_210 group was much higher than that in the 30_vs_90 and 90_vs_210 groups, consistently with the differences in phenotype. According to the expression level at each developmental stage, the DEGs were grouped into eight clusters. Among these, the expression levels of the genes in clusters 6 and 8 increased with age, and these genes were mainly enriched in BP terms associated with muscle development, as revealed by GO enrichment. The results provide genetic data that can be used to further reveal the mechanisms underlying muscle development in Min pigs.

Through an ATAC-seq and RNA-seq integrated analysis, 54 genes were identified as key regulators of muscle development in Min pigs, as they were not only differentially expressed but differentially accessible in the promoter, that is, the DEG_DAR_gene; additionally, most of these genes, 48 of them, were found in the 30_vs_210 group. The 48 genes, including *SLPI*, *APOA1*, *SLA*, and *PARVG*, are associated with various biological processes, such as cell proliferation, the immune response, and metabolism [44,45,46]. Through an ATAC-seq and RNA-seq integrated analysis, TFs regulating the DEG_DAR_genes were identified. Among the TFs shared between all three comparison groups, KLF5 and ZNF148 had the most target genes, each of which were targeted to the promoter of 36 DEG_DAR_genes. Compared with ZNF148, the expression changes in KLF5 were robust among the three comparison groups. Thus, the regulatory role of KLF5 in the DEG_DAR_genes was identified using molecular biology techniques.

In this study, the promoter regions were defined as 3 kb regions upstream and downstream of the TSSs; thus, TFs were predicted not only in the promoter regions but in the DARs downstream of the gene. Peaks 7 and 1, located in the promoter of *SLPI* and downstream of *APOA1*, respectively, were then selected for further analysis. Through a series of molecular experiments, including a dual-luciferase reporter gene analysis, site-directed mutagenesis of the putative motif, the ectopic expression of KLF5, qPCR, EMSA, and ChIP-qPCR analyses, it was made clear that KLF5 regulated the expression of *SLPI* by binding to the promoter region.

Next, the role of KLF5 in *APOA1* expression was preliminarily analyzed using the dual-luciferase reporter gene and overexpression technology, and the results show that the putative motifs of KLF5 were active and that KLF5 may play a role in *APOA1* expression. TFs were identified as binding to the motif in the promoter to regulate gene transcription. Recently, increasing evidence has shown that the TF binding sites are diverse. In addition to the promoter regions, DNA in the intron regions and transcription end sites, RNA sequences, and even RNA–DNA hybrids are all potential sites for *trans*-acting factor recognition [47,48,49]. Furthermore, it has been suggested that, in addition to transcription regulation, TFs play multiple roles in RNA processing, cleavage, and polyadenylation. For example, some Cys2His2-zinc-finger proteins, such as CTCF, YY1, and TFIIIA, were found to directly bind RNA, as well as DNA [50,51,52,53]. Sp1 was found to bind to RNA 3′ UTRs directly by using its zinc-finger domains and repress the usage of distal poly(A) sites, which enabled Sp1 to regulate mRNA levels by controlling 3′ UTR length and, thus, mRNA stability [49]; conversely, CTCF was demonstrated to recruit the cohesion complex to generate chromatin loops, which enhanced the usage of proximal poly(A) sites [53]. These results revealed that Sp1 and CTCF play roles independent of TFs. Here, through overexpression and dual-luciferase reporter gene assays, we found that KLF5, located in intron 3 of the *APOA1* gene, can act on the motifs of intron 3 to regulate *APOA1* expression, suggesting that it plays a novel role independent of TF binding to the promoter. However, more experiments are needed to support this suggestion. We will further endeavor to determine whether KLF5 can directly bind to the putative motif and to reveal the mechanisms through which KLF5 regulates *APOA1* expression. Nevertheless, we confirmed that the TFs identified here are reliable and that KLF5 is a TF of *SLPI*.

## 5. Conclusions

In this study, by using ATAC-seq and RNA-seq, we investigated the global landscape of chromatin accessibility and the transcriptome in the skeletal muscle of Min pigs at three developmental stages. Chromatin accessibility and the transcriptome changed dynamically with muscle development. A total of 16,301 DARs, associated with 7455 genes and 2219 DEGs, were identified. A sequential analysis showed that the expression of genes involved in muscle formation continuously increased as development progressed. Importantly, through integrated analyses of ATAC-seq and RNA-seq data, 54 DEG_DAR_genes and 61 TFs were identified as critical for muscle development. Furthermore, by using KLF5 as an example with molecular biology techniques, it was revealed that the regulatory role of TFs in the DEG_DAR_genes was reliable. KLF5 not only regulates the expression of *SLPI* by directly binding to the promoter but exerts an effect on the regulatory role of DAR located in the intron of *APOA1*. To sum up, we characterized the TFs and genes important for muscle development in Min pigs. The results provide genetic data for further revealing the mechanisms underlying muscle development in pigs.

## Data Availability

The data that support this study are provided in the main text or the Supplementary Materials. All raw sequencing data have been deposited in the Genome Sequence Archive in National Genomics Data Center, China National Center for Bioinformation/Beijing Institute of Genomics, Chinese Academy of Sciences (GSA: CRA013591 for RNA-seq data, submitted on 24 October 2024; CRA019293 for ATAC-seq data, submitted on 27 September 2024) that are publicly accessible at https://ngdc.cncb.ac.cn/gsa.

## Supplementary Materials

The following supporting information can be downloaded at: https://www.mdpi.com/article/10.3390/cells13242118/s1, Figure S1: GO terms enriched by genes in cluster 2; Table S1: Oligonucleotide sequences; Table S2: Statistics of ATAC-seq data; Table S3: Differential peaks and associated genes; Table S4: Detailed information for eight peaks; Table S5: Differentially expressed genes; Table S6: Common genes between DEGs and DAR associated genes; Table S7: DEGs containing DARs in the promoter; Table S8: TFs predicted in DEGs containing DARs in the promoter.
