# Supplementary material for: Integrative Analysis of Chromatin Accessibility and Transcriptional Landscape Identifies Key Genes During Muscle Development in Pigs
Source: Cells. 2024 Dec 20;13(24):2118. doi: 10.3390/cells13242118 (PMC11727100; doi:10.3390/cells13242118)
Supplement: Supplementary file 1 [file cells-13-02118-s001.zip › Figure S GO terms enriched by genes in cluster 2.pdf]

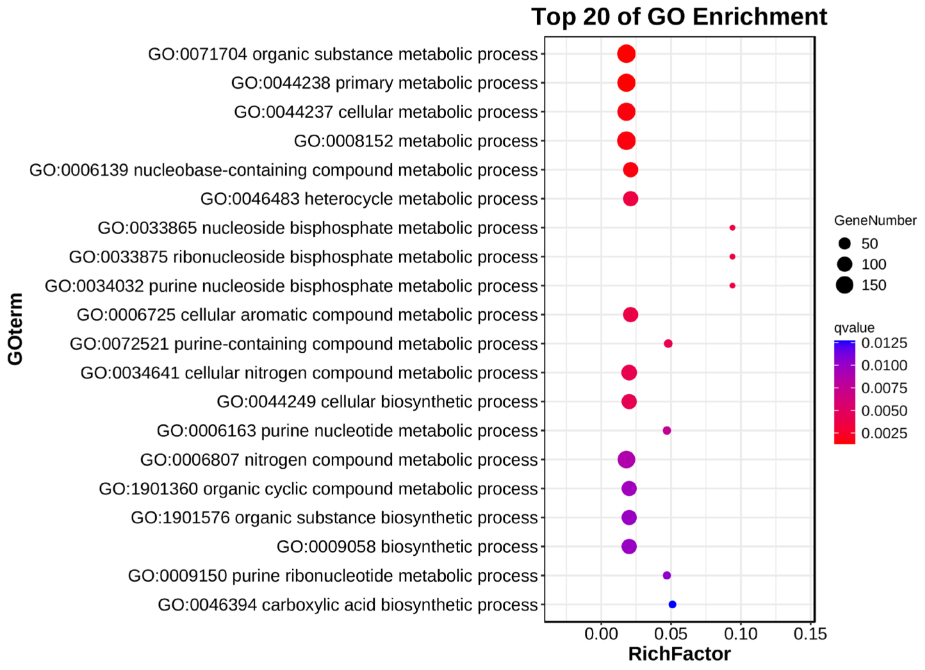

Figure S1 Top 20 biological process terms enriched by genes in cluster 2 as revealed by GO enrichment.
