# Supplementary material for: Integrative Analysis of Chromatin Accessibility and Transcriptional Landscape Identifies Key Genes During Muscle Development in Pigs
Source: Cells. 2024 Dec 20;13(24):2118. doi: 10.3390/cells13242118 (PMC11727100; doi:10.3390/cells13242118)
Supplement: Supplementary file 1 [file cells-13-02118-s001.zip › Table S1 Oligonucleotide sequences.pdf]

Table S1-1 Primers used for plasmid construction

| Names | Sequences (5'-3') | Purposes |
|---|---|---|
| Peak1-XbaI-F | AGATCGCCGTGTAATTCTAGACAGACAGGACTGGGCAC | |
| Peak1-XbaI-R | GCCGGCCGCCCCGACTCTAGACAACCCATCACAAGCGTC | |
| Peak2-Hind3-F | ATCTGCGATCTAAGTAAGCTAGGAGGTAAAGCACCAGCT | |
| Peak2-Hind3-R | CAGTACCGGAATGCCAAGCTTTGCGACACACTGGAGAC | |
| Peak3-Hind3-F | ATCTGCGATCTAAGTAAGCTGATGAAAGACGATGCAGTTATG | |
| Peak3-Hind3-R | CAGTACCGGAATGCCAAGCTCATCACATTGACTTGGGAGAC | |
| Peak4-XbaI-F | AGATCGCCGTGTAATTCTAGAGCCATGTGTGTTGGGGGAT | |
| Peak4-XbaI-R | GCCGGCCGCCCCGACTCTAGACCCCAGATGAAGGCAACTGA | |
| Peak5-Hind3-F | ATCTGCGATCTAAGTAAGCTAGCTGCTCTTCCGCGTCCCT | Luciferase reporter |
| Peak5-Hind3-R | CAGTACCGGAATGCCAAGCTCCTTCCCCGATTCCTCA | gene construction |
| Peak6-Hind3-F | ATCTGCGATCTAAGTAAGCTTAAAAGTATGAATGTAAAGAGC | |
| Peak6-Hind3-R | CAGTACCGGAATGCCAAGCTCAAGATTTTTCAACCAGTG | |
| Peak7-Hind3-F | ATCTGCGATCTAAGTAAGCTTTGTCCAGGGCTGAGGT | |
| Peak7-Hind3-R | CAGTACCGGAATGCCAAGCTCCATTTTCAGCACCTTCCACGG | |
| Peak8-Hind3-F | ATCTGCGATCTAAGTAAGCTTGATGGCAACAGAACCTCAA | |
| Peak8-Hind3-R | CAGTACCGGAATGCCAAGCTTTTTTTCAACCAGTGAAAG | |
| Peak1-J1-F | AGATCGCCGTGTAATTCTAGACCACACTTTCAGGGGCAC | 5' truncation of |
| Peak1-J2-F | AGATCGCCGTGTAATTCTAGATTGCAGCTCCAGAGGTG | Peak1 |
| 3HA-F | TGCATGGTACCCGGGTACCCGTACGACGTCCCG | Construction of |
| 3HA-KLF5-R3 | GCGTAGCCATGGCATAATCTGGAACATCG | plasmids |
| 3HA-KLF5-F3 | GATTATGCCATGGCTACGCGGGTGC | overexpressing |
| KLF5-PcAGGs-R | TCGAGCATGCCCGGGTCAGTTCTGGTGCCTCTTCATG | KLF5 |
| Peak7-KLF5-F | TGCCTGTGAAGAGTGAAGGTATAAACAG | Deleting KLF5 |
| Peak7-KLF5-R | ACCTTCACTCTTCACAGGCATTTCCT | motif |

Table S1-2 Primers used for real-time quantitative PCR

| Names | Sequences (5'-3') |
|---|---|
| SESN1-F | CGACCACTAGGACACGGA |
| SESN1-R | ACCCAAAGCAGCAAAAGA |
| ATP5F1A-F | GAGCCTAGCAAGATCACAAA |
| ATP5F1A-R | CTTCAAATCCAGCCAAGAAG |
| PSMC6-F | TGAAAGCAGTCAGAAAAGTGG |
| PSMC6-R | TGTCATGCAGCCATCAAAAA |
| RAD23B-F | CTGGAGATAGAGAAAGTCAG |
| RAD23B-R | CGTAAAAATTCAAGAGGATG |
| RSP7-F | GGCTGCCAAAAACTCATTGA |
| RSP7-R | TTCGGACCACATAACCCTTC |
| CD164-F | AAACCACAACTCTGCCTTCC |
| CD164-R | AAATTACAGCCTGCACACCC |
| HSPA5-F | ACCTTCTGGGAACTTTTGAT |
| HSPA5-R | TTTGTTGCCTGTACCTTTGT |
| EIF3J-F | AAGGTGTGGTTCCTGGAGGG |

| | |
|---|---|
| EIF3J-R | ATGTTGAAGGGATTGTGGGC |
| ZFAND5-F | TCTCAAAGTGAAGAAAAAGC |
| ZFAND5-R | GTCAGAGTAACGGTGAAGTC |
| OAZ2-F | AAACCTCACATCGTCCACTT |
| OAZ2-R | CTCCCATCAGCTAATAATCC |
| APOA1-F | CCTCCGCTTTGGGAAAACACCT |
| APOA1-R | TCGGTCTCCTTTTCCAGGTTGT |
| SLPI-1 | CAAGTGCACAAGTGACTGGC |
| SLPI-2 | GGCCATAGACCACTGGACAC |

Table S1-3 Oligonucleotides used for Electrophoretic mobility shift assay

| Names | Sequences (5’-3’) |
|---|---|
| biotin-labeled | F: bio-GTGAAGCCACACCCACGAGTGAAGG |
| | R: bio-CCTTCACTCGTGGGTGTGGCTTCAC |
| Specific competitor | F: GTGAAGCCACACCCACGAGTGAAGG |
| | R: CCTTCACTCGTGGGTGTGGCTTCAC |
| Mutant competitor | F: GTGCCCGGTAAGTAGACTGCCAAGG |
| | R: CCTTGGCAGTCTACTTACCGGGCAC |
